# Bicarbonate exchangers SLC26A3 and SLC26A6 are localized at the apical membrane of porcine vas deferens epithelium

**DOI:** 10.14814/phy2.12380

**Published:** 2015-04-23

**Authors:** Fernando Pierucci-Alves, Vladimir Akoyev, Bruce D Schultz

**Affiliations:** Department of Anatomy & Physiology, Kansas State UniversityManhattan, Kansas, USA

**Keywords:** Epithelia, ion transport, vas deferens

## Abstract

The goal of this study was to test for expression of HCO_3_^−^ exchangers SLC26A3 and SLC26A6 in primary cultures of porcine vas deferens epithelial cells (1°PVD) and native porcine vas deferens. Quantitative RT-PCR revealed that mRNA coding for SLC26A6 was six times more abundant than mRNA coding for SLC26A3 in 1°PVD cells. Western blot analyses combined with surface biotinylation of 1°PVD demonstrated SLC26A3 and SLC26A6 immunoreactivities in whole-cell lysates and apical surfaces of monolayers. Laser scanning confocal microscopy (LSCM) of the 1°PVD cell monolayers demonstrated that SLC26A3 immunoreactivity was primarily in the apical region but present throughout the basal-apical cellular axis, whereas SLC26A6 immunoreactivity was present in the apical region and sometimes accumulated in the nuclear region. LSCM also demonstrated SLC26A3 and SLC26A6 immunoreactivities present along the entire apical lining of the native porcine vas deferens epithelium and in basal cells. The patterns and apparent abundance of SLC26A3 and SLC26A6 immunoreactivities in the proximal vas deferens were not different from the corresponding immunoreactivities in the distal region. There is no evidence of preferential expression of SLC26A3 or SLC26A6 in any portion of the vas deferens, as has been proposed for epithelia that secrete HCO_3_^−^ in other duct systems. Thus, vas deferens epithelia express transporters throughout the duct that can contribute to rapid alkalinization of the luminal contents as it has been demonstrated *in vivo*.

## Introduction

Human patients with mutations in both *SLC26A3* alleles present with infertility that is associated with absence of SLC26A3 (Cl^−^-losing diarrhea, CLD) expression in the efferent ducts, oligoasthenoteratozoospermia, high Cl^−^ concentration and low pH in seminal plasma, and spermatoceles (Hihnala et al. 2006; Hoglund et al. 2006). The efferent ducts drain fluid and sperm from the rete testis and constitute the most proximal portion of the male excurrent duct, which is subsequently comprised by the epididymis and vas deferens. Specialized epithelia line this duct system, carrying out a number of absorptive and secretory mechanisms that are required for sperm maturation, acquisition of fertilizing capacity and fertility (Bedford 1973). Moreover, membrane 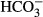 transport mechanisms are known to be present and relevant for these physiological processes (Liu et al. 2012; Bernardino et al. 2013). SLC26A3 is a Cl^−^/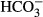 exchanger and low pH in seminal plasma of CLD patients suggests that SLC26A3 is a major participant in 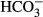 secretion onto the lumen of the male excurrent duct and/or male accessory glands. Moreover, SLC26A6 (also known as putative anion transporter-1, PAT-1 or Cl^−^-formate exchanger, CFEX) is reported to transport oxalate and sulfate in addition to Cl^−^ and 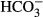, and thus may also have a unique physiological role in the male reproductive duct. Expression of SLC26A6 has been reported in a variety of tissues including kidney and pancreatic duct although no disease has yet been associated with naturally occurring *SLC26A6* mutations. *Slc26a6* knockout mice exhibit a generally mild phenotype that includes calcium oxalate urolithiasis (Wang et al. 2005; Jiang et al. 2006), however, a reproductive phenotype has not been reported. Importantly, both SLC26A3 and SLC26A6 are reportedly co-regulated by or with the cystic fibrosis transmembrane conductance regulator (CFTR) in *in vitro* expression systems (Ko et al. 2002). Thus, male reproductive duct destruction (i.e., congenital bilateral atresia of the vas deferens; CBAVD) (Bedford 1973), which is observed in most cystic fibrosis patients, and other forms of infertility or subfertility associated with CFTR mutations may reflect aberrant regulation of one or both of these anion exchangers.

Our laboratory developed and employs a porcine vas deferens epithelial cell model to study transport mechanisms. Employing this model, we have shown that anion secretion includes both 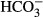 - and Cl^−^-dependent components (Sedlacek et al. 2001; Carlin et al. 2002), and can be stimulated by adrenergic and purinergic neurotransmitters (Sedlacek et al. 2001; Carlin et al. 2003, 2006), peptide hormones (Hagedorn et al. 2007) and autacoids (Pierucci-Alves and Schultz 2008). Cells derived from the distal portion of the human vas deferens have a similar profile of anion secretion regulated by physiological and pharmacological agents (Carlin et al. 2003; Hagedorn et al. 2007; Pierucci-Alves and Schultz 2008). We proposed that the release of neurotransmitters such as norepinephrine during the preejaculatory arousal period would be expected to induce 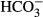 secretion *in vivo* that would raise luminal pH in the vas deferens and initiate sperm activation (Sedlacek et al. 2001). This hypothesis is supported by the report that porcine vas deferens luminal pH is neutral *in vivo* and rises rapidly after systemic adrenergic stimulation (Pierucci-Alves et al. 2010). However, ion transporters to account for 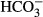 secretion across vas deferens epithelium have not been fully defined. Our data support that CFTR is an apical membrane 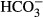 exit route (Sedlacek et al. 2001; Carlin et al. 2002, 2006). PVD9902 cells, an immortalized porcine vas deferens epithelial cell line, expresses mRNA coding for both SLC26A3 and SLC26A6 (Carlin et al. 2006). However, data to support protein expression and localization of these ion exchangers are not available.

The goal of this study was to determine whether the 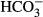 exchangers SLC26A3 and SLC26A6 are expressed in primary cultures of porcine vas deferens epithelia and/or native cells lining the intact boar vas deferens. Experiments were designed specifically to determine whether either of these transporters is expressed in distinct cell populations and to determine whether the general pattern of expression is uniform throughout the vasa. Immunochemistry and indirect immunofluorescence provide evidence that both SLC26A3 and SLC26A6 are expressed widely throughout the porcine vas deferens although some differences in subcellular distribution are reported. The observations suggest that SLC26A3 and/or SLC26A6 can contribute to the rapid alkalinization of the vas deferens lumen that has been reported following systemic adrenergic stimulation (Pierucci-Alves et al. 2010).

## Materials and Methods

### Tissue acquisition

Vas deferens from sexually mature boars (∼6 months old) were acquired at a local swine production facility immediately post mortem as described in detail previously (Sedlacek et al. 2001). Segments (∼1 cm) were derived from proximal and distal ends of the vas deferens during dissection and snap frozen in liquid nitrogen for subsequent immunohistochemistry and confocal microscopy.

### Epithelial cell isolation and culture

Porcine vas deferens epithelial cells (1°PVD) were isolated from ducts and cultured as described previously in detail (Sedlacek et al. 2001). Cells isolated from each duct were seeded on 25 cm^2^ tissue culture flasks (Corning, Inc., Corning, NY) and grown in Dulbecco modified Eagle's medium, (Invitrogen, Baltimore, MD) supplemented with 10% fetal bovine serum (Atlanta Biologicals, Atlanta, GA), penicillin (100 U/mL), and streptomycin (100 *μ*g/mL; Invitrogen). After 4–6 days in culture, cells were suspended and seeded on permeable supports (Transwell or Snapwell, Corning, Inc.). Cells grew as confluent monolayers and were fed every other day until used for experiments, typically 14–16 days.

### RNA isolation and quantitative RT-PCR

RNA was isolated via the phenol–chloroform extraction method from 1°PVD epithelial cell monolayers. RNA samples were subjected to DNase treatment according to manufacturer's recommendations (Bio-Rad, Hercules, CA), and concentration and quality were assessed spectrophotometrically (ND-1000, NanoDrop Technologies, Wilmington, DE) and with microfluidics (2100 Bioanalyzer, Agilent Technologies, Santa Clara, CA), respectively. All RNA samples were then normalized to 250 ng/*μ*L. Subsequently, absolute quantitation of the porcine *SLC26A3* and *SLC26A6* transcripts in each RNA sample was conducted via an assay that included a cDNA standard curve. In brief, primer sets selective for the targeted transcripts that have been characterized and reported previously (Carlin et al. 2006) were employed in RT-PCR reactions to synthesize the respective cDNAs. cDNA samples were then purified with a DNA purification system (Qiagen, Inc., Valencia, CA) and spectrophotometrically quantified. Based on the estimated molecular weight of each cDNA template and the cDNA sample concentration, serial dilutions were made to achieve cDNA templates at predetermined copy numbers that were added to reactions composing the standard curve in each assay. RT-PCR reactions employed the One-Step RT-PCR reagent kit (Qiagen, Inc.), and were conducted in duplicates using 500 ng of total RNA per reaction. Five unique RNA samples (derived from five individual 1°PVD isolations) were employed in this assay. All reactions targeted a single template and amplification was detected via SYBR-green fluorescence following each reaction cycle (iCycler IQ, Bio-Rad). The amplified porcine SLC26A6 sequence shares homology with a segment of human SLC26A6 that is common to all human transcript variants described to date. Gel electrophoresis was performed with all reaction products to verify that the mobility of each amplicon was as expected. cDNA copy numbers present in each standard curve step and the respective threshold cycle (*C*_t_) value were employed to determine, by linear regression, the transcript copy numbers in each RT-PCR reaction, via their respective *C*_t_ values.

### Surface protein biotinylation

General protocols for optimizing and conducting surface biotinylation labeling assays have been described previously (Gottardi and Caplan 1992; Gottardi et al. 1995). Briefly, 1°PVD epithelial cells derived from each of four boars were cultured as confluent monolayers on permeable supports in two 6-well Transwell plates (12 inserts, 4.67 cm^2^ per insert). All monolayers were washed free of serum proteins in three changes of Ringer solution (composition in mmol/L: 120 NaCl, 25 NaHCO_3_, 3.3 KH_2_PO_4_, 0.83 K_2_HPO_4_, 1.2 CaCl_2_, 1.2 MgCl_2_) and then incubated in Ringer solution at 37°C for 30 min. Monolayers were gradually cooled to ∼4°C on ice for 30 min followed by exposure to vehicle or biotin (EZ-link® Sulfo-NHS-LC-Biotin, Pierce, Rockford, IL) either apically or basolaterally (three wells, each) for 30 min with gentle agitation. At the end of the incubation period, apical and basolateral solutions were removed and cells were washed five times (3 min each) with 50 mmol/L glycine in Ringer solution. Cells were then harvested by scraping into phosphate-buffered saline (PBS) and pelleted at 600g for 5 min at 4°C. Pellets were lysed in RIPA buffer (1.0 mL per sample) supplemented with protease inhibitors (Roche Complete Mini, Roche, Indianapolis, IN), passed 10 times through a 30 G needle, and sedimented at 20,200 *g* for 15 min at 4°C. Supernatant was collected and considered as whole-cell lysate (WCL). Lysates from identically treated wells were combined for separation, resolution, and analysis. Protein concentration was determined using the bicinchoninic acid assay (Micro BCA Protein Assay Kit, Pierce) and the concentration for all samples was adjusted to 2 mg/mL with RIPA buffer. Resin for separation (NeutrAvidin, Pierce) was placed into spin columns (0.5 mL per sample) and washed three times with 0.5 mL RIPA buffer. WCL (0.5 mL) was added to the resin, mixed carefully and incubated for one hour at room temperature under constant rotation. After incubation, each sample was separated by centrifugation and the eluent was retained for further analyses. The resin was washed several times with RIPA buffer (plus protease inhibitors) in the same spin columns until absorbance at 280 nm could not be detected in the eluent (typically four washes of 0.5 mL). Avidin-bound proteins were eluted by adding 0.5 mL of Laemmli sample buffer with 2% SDS and 50 mmol/L dithiothreitol followed by incubation for one hour at room temperature and constant rotation. Eluents were collected by centrifugation.

### Western blots

Electrophoresis of protein samples was conducted using 4–20% gradient Tris-HEPES-SDS precast mini gels (Thermo Scientific, Rockford, IL). After electrophoresis, gels were soaked three times, 10 min each, in transfer buffer (192 mmol/L glycine, 25 mmol/L Tris base, and 10% methanol) to remove SDS and to prevent gel shrinkage. Proteins were transferred to polyvinylidene difluoride membranes (Immobilon-P 0.45 *μ*m, Millipore, Bedford, MA) for 4 h under 2 mA/cm^2^ at 4°C. After transfer, membranes were blocked with 5% dry milk in PBS overnight. Membranes were then incubated with primary antibody for 12 h at 4°C. After washing with PBS-Tween buffer (0.1% Tween 20 in PBS, pH 7.5; five washes, 5 min, each), membranes were exposed to a suitable horseradish peroxidase (HRP)-conjugated secondary antibody (Thermo Scientific). Membranes were then incubated with SuperSignal West Femto Chemiluminescent Substrate (Pierce). Gel images were captured digitally (Imagestation 4000R, Eastman Kodak Co., Rochester, NY). To detect SLC26A3 or SLC26A6 on immunoblots, 1 *μ*g/mL of primary antibody and 10 ng/mL of HRP-conjugated IgG were employed.

### Cryosection preparations for confocal microscopy

Snap-frozen tissue samples of proximal and distal vas deferens (∼1 cm) derived from seven boars were examined. Frozen proximal and distal segments were placed together, vertically, into the same mold, embedded (Cryogenic-Gel; Electron Microscopy Sciences, Hatfield, PA), and sectioned for immunohistochemistry (10 *μ*m; Leica Microsystems, Bannockburn, IL). In order to process and analyze sections from different boars in the same conditions, sections from as many as five different boars were placed together on the same slide. Mounted sections were dried under constant air flow for 12 h at room temperature. Sections were dehydrated by dipping in acetone (−20°C) followed by drying for 1 h with constant air flow at room temperature. Fixation was completed in acetone:methanol (9:1) on dry ice (−60°C) for 15 min. After fixation, sections were allowed to dry for 5 h under air flow at room temperature. Sections were rehydrated in PBS and incubated in the blocking solution (5% BSA, 0.1% Tween-20, in PBS, pH 7.4) for 36–48 h at 4°C and constant shaking. Blocking solution was changed every 6–8 h. Then sections were subjected to dual fluorescence labeling with two primary antibodies: anti-occludin (3 *μ*g/mL) with either anti-SLC26A3 (4 *μ*g/mL) or anti-SLC26A6 (4 *μ*g/mL) in the blocking solution for 12 h at 4°C and constant shaking. Sections were washed with PBS-0.1% Tween-20 buffer using a squirt bottle (∼100 mL per slide) followed by incubation with two secondary antibodies conjugated to Alexa Fluor 488 or Alexa Fluor 594 (15 ng/mL) in the blocking solution for 2 h at room temperature in the dark. Sections were washed with PBS (∼100 mL per slide) and incubated in 300 nmol/L DAPI (Invitrogen) in PBS for 1 h. Sections were mounted with FluorSave™ reagent (Calbiochem, EMD, San Diego, CA) and glass cover slips, and allowed to dry for a minimum of 2 h at room temperature before slides were assessed by confocal microscopy.

### Epithelial cell monolayer preparation for confocal microscopy

1°PVD cells cultured on permeable supports were fixed for 5 min in PBS containing 4% paraformaldehyde (Electron Microscopy Sciences). Paraformaldehyde was quenched with three 5-min washes with 50 mmol/L glycine in PBS. Monolayers were washed with PBS, permeabilized with 0.5% Triton X-100 in PBS for 30 min, washed with PBS, and incubated in the blocking solution for 48 h at 4°C. After blocking, cell monolayers on filter supports were cut into several pieces that were subjected to one of three general protocols: (1) dual immunolabeling with anti-occludin (3 *μ*g/mL) and either anti-SLC26A3 (10 *μ*g/mL) or anti-SLC26A6 (10 *μ*g/mL); (2) dual immunolabeling as in 1, but with SLC26A-antibodies that had been preadsorbed with their respective antigenic peptides; or (3) exposed to Alexa Fluor secondary antibodies (20 *μ*g/mL) only. Cells were incubated with primary antibodies in the blocking solution for 12 h at 4°C*,* washed five times with PBS, and incubated with two Alexa Fluor-conjugated secondary antibodies in the blocking solution for 2 h at room temperature in the dark. Monolayers were washed with PBS and incubated with DAPI (300 nmol/L in PBS) for 1 h. Cell monolayers were mounted on slides, covered by FluorSave™ reagent and sealed with cover glass. After FluorSave polymerization was completed, monolayers were used for confocal analysis.

### Scanning confocal microscopy

Images were acquired with a Zeiss LSM 510 confocal microscope and a 40 × 1.3 NA plan-NEOFLUAR oil differential interference contrast objective lens with appropriate excitation wavelengths for DAPI, Alexa Fluor 488, and Alexa Fluor 594. Pinholes were set to one Airy unit for each laser line at which axial resolution was 533 nm for *λ*_em_ 450 nm, 614 nm for *λ*_em_ 518 nm, and 732 nm for *λ*_em_ 617 nm. Corresponding optical slice thicknesses were 700 nm, 900 nm, and 1 *μ*m, respectively. In the case of 10 *μ*m tissue sections, series of images (z-stacks) were collected along 15 *μ*m in the z-direction with sampling rates of 110, 110, and 370 nm/pixel along the x-, y-, and z-axis, respectively. In the case of cultured epithelial monolayers, images were collected along 15 *μ*m in the z-direction with a Nyquist sampling rate of 40×40×165 nm/pixel (x, y, z) (Nyquist Online Calculator, 2009 Scientific Volume Imaging b.v., http://support.svi.nl/wiki/NyquistCalculator). Intensities of 488-Alexa Fluor and 594-Alexa Fluor signals (without primary antibodies) were estimated by acquiring images of monolayers or sections using the same microscope settings at which fluorescence with primary antibodies were acquired. Images of monolayers labeled with a single antibody were acquired and analyzed to insure that no bleedthrough between channels could be detected. The amount of ‘noise’ in a region of interest was estimated by measuring the Pearson correlation coefficient of two consecutive acquisitions of the same channel. Autofluorescence of 1°PVD sections was detected only at maximum gain and was not detected at the gain level used for image acquisition. Image analysis was performed with public domain software, ImageJ (http://rsb.info.nih.gov/ij/).

### Semiquantitative image analyses

The distribution of the fluorescent signal (immunoreactivity) in 1°PVD cells was analyzed by counting voxels per slice in z-stacks. Analysis was carried out using ImageJ software with the “Voxel Counter” plug-in (http://rsb.info.nih.gov/ij/plugins/voxel-counter.html). 1°PVD monolayers from four cell isolations were analyzed. At least two monolayers per isolation were evaluated. Monolayers on filter supports were cut into several small pieces (∼3 × 3 mm) for processing and samples were processed in parallel. Several images per monolayer were acquired at random using identical confocal settings and three were selected for voxel counting. Monolayer images were collected along 15 *μ*m of the z-axis as described above. The focal plane with the highest intensity of anti-occludin fluorescence was employed as the benchmark to standardize the region of data acquisition. Optical slices were acquired throughout the region from at least 5 *μ*m above the benchmark to at least 10 *μ*m below the benchmark slice. Optical stacks of background signals were collected outside the membrane area using the same microscope settings and background images were subtracted from monolayer images prior to analysis.

Semiquantitative analyses of epitope abundance and distribution were conducted using a stepwise procedure to normalize the data sets. For each optical channel, the fluorescent value for each voxel was converted to binary output (fluorescence present or absent) using a threshold intensity of 50 fluorescent units (0–255 range). The optical slice with the greatest number of positive voxels was used to normalize all other planes and the results are reported as percent of this maximum value.

### Antisera

All primary antibodies employed in this study were commercially available. Initially, various antibodies for SLC26A3 and SLC26A6 were purchased and tested against epithelial and nonepithelial cell lines, mostly of porcine and human origin. As a result of these antibody validation studies, goat polyclonal anti-human-N-terminus SLC26A3 antibody (Santa Cruz Biotechnologies, Santa Cruz, CA, cat. 34942) and goat polyclonal anti-human-C-terminus SLC26A6 antibody (Santa Cruz Biotechnologies, cat. 26728), along with their respective immunizing peptides (cats. 34942P and 26728P) were chosen and employed throughout the experiments reported here. Other primary antibodies employed were mouse monoclonal anti-human-C-terminus occludin antibody (Invitrogen, cat. 33-1500), and rabbit anti-C-terminus actin antibody (Sigma, cat. A2066). Alexa Fluor 488- and 594-conjugated antibodies (Invitrogen, Carlsbad CA) of suitable species-reactivity were employed for detection.

### Primary antibody preadsorption/competition assays

In some experiments, the anti-SLC26A antibodies were preadsorbed by their respective immunizing peptide for 24 h at 4°C in blocking solution (5% nonfat dry milk-Tween for western blot and 5% BSA-Tween-PBS for immunocytochemistry). In each of the various assays, the mass concentration of the immunizing peptide was 50 times that of the primary antibody. After incubation, solutions were sedimented at 20,200 *g* for 15 min at 4°C and supernatants were used for blotting or immunocytochemistry.

## Results

### mRNAs coding for SLC26A3 and SLC26A6 are present in 1°PVDs

**Figure 1:**
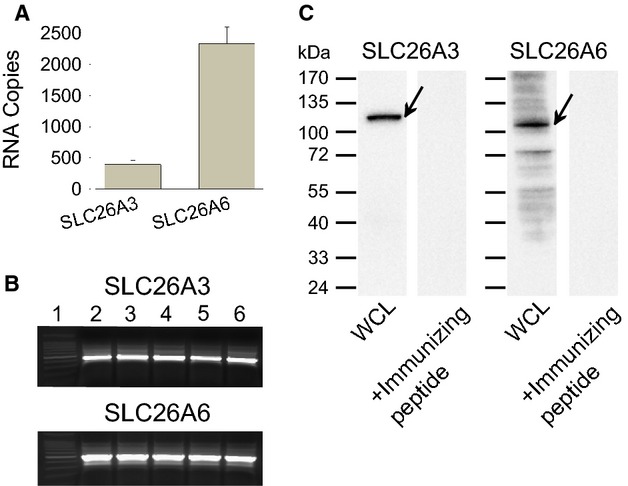
Primary cultures of porcine vas deferens epithelial (1°PVD) cells express HCO_3_^−^ transporters. (A) Number of mRNA copies coding for SLC26A3 and SLC26A6 in RNA isolated from 1°PVD cells. (B) Gel images demonstrate a single amplicon with expected mobility following single target qRT-PCR conducted with primers for SLC26A3 (424 bp) and SLC26A6 (494 bp). Lane 1, DNA ladder; lanes 2–6, RT-PCR products derived from five RNA samples that were isolated from 1°PVD monolayers (five different animals). (C) Western blot analyses reveal SLC26A3 and SLC26A6 immunoreactivities in whole-cell lysates (WCL) derived from 1°PVDs. Blots were probed with 0.5 *μ*g/mL of anti-SLC26A3 antibody or anti-SLC26A6 antibodies (WCL), or probed with antibodies preadsorbed to immunizing peptide. Arrows indicate major bands detected. Results are representative of six 1°PVD monolayers (six different animals).

Initial experiments were conducted to quantify SLC26A3 and SLC26A6 transcripts in RNA isolated from 1°PVD cells grown as polarized confluent monolayers. Prior to RNA isolation, monolayers were voltage-clamped in Ussing chambers and exhibited basal transepithelial resistance and responsiveness to forskolin in magnitudes similar to data reported previously (Sedlacek et al. 2001; Carlin et al. 2002; Phillips and Schultz 2002). qRT-PCR targeting SLC26A3 or SLC26A6 produced single melting peaks and bands of expected mobility. Amplification curves from RT-PCR reactions generated mean *C*_t_ values of 32.34 ± 0.90 and 27.62 ± 0.35 for SLC26A3 and SLC26A6, respectively. Figure1A depicts the copy number of each transcript present in 500 ng of total RNA. Images in Figure1B demonstrate that a single product of expected mobility was generated in each reaction. These data demonstrate clearly that RNAs coding for both SLC26A3 and SLC26A6 are expressed by 1°PVDs and that there are nearly six times more copies of SLC26A6 mRNA than that of SLC26A3.

### SLC26A3 and SLC26A6 immunoreactivities are present in 1°PVD lysates

To determine whether SLC26A3 and SLC26A6 protein immunoreactivities are present in 1°PVDs, western blot analyses were conducted. SLC26A3 immunoreactivity in whole 1°PVD lysates was detected as a single band that exhibited mobility consistent with ∼120 kDa (Fig.1C). Porcine SLC26A3 should contain 759 amino acids and a theoretical molecular weight of 84 kDa. This ∼120 kDa mobility detected here is consistent with those reported previously by others (Dorwart et al. 2008; Hayashi et al. 2009) and with our own previous work (Pierucci-Alves et al. 2011). *N*-glycosylation of SLC26A3 was shown to account for a molecular weight increase from an expected ∼80 kDa to a detected ∼120 kDa (Hayashi et al. 2009). *N*-glycosylation is significant and prevalent among most member of the SLC26A family (Li et al. 2014). Moreover, the anti-SLC26A3 employed here was tested against whole-cell lysates derived from T84 and LLC-PK_1_ monolayers paired to 1°PVDs and a single band of the same mobility (∼120 kDa) was detected in all experimental groups (data not shown). No SLC26A3 immunoreactivity was observed when the antibody was preadsorbed by incubation with its immunizing peptide for 24 h (Fig.1C).

SLC26A6 immunoreactivity was also detected in 1°PVD cell lysates in the form of a major band with mobility indicating molecular mass of ∼110 kDa, along with a set of minor bands exhibiting greater mobility (Fig.1C). The apparent mass of the major band was greater than the theoretical mobility expected for porcine SLC26A6 (753 amino acids; ∼82 kDa), likely due to *N*-glycosylation (Li et al. 2014). Additional results we acquired suggesting that the ∼110 kDa band is specific were that this same signal was present when the anti-SLC26A6 was tested against T84 and LLC-PK_1_ lysates (data not shown). This same ∼110 kDa signal was detected and reported by us previously while using the same anti-SLC26A6 against lysates from cultured and native neonatal porcine vas deferens (Pierucci-Alves et al. 2011). Moreover, specificity of the anti-SLC26A6 was also supported by results from preadsorption assays (Fig.1C). All together, these data suggest that SLC26A3 and SLC26A6 are expressed at the protein level in 1°PVD.

### Patterns of SLC26A3 and SLC26A6 distributions in 1°PVDs are distinct

**Figure 2:**
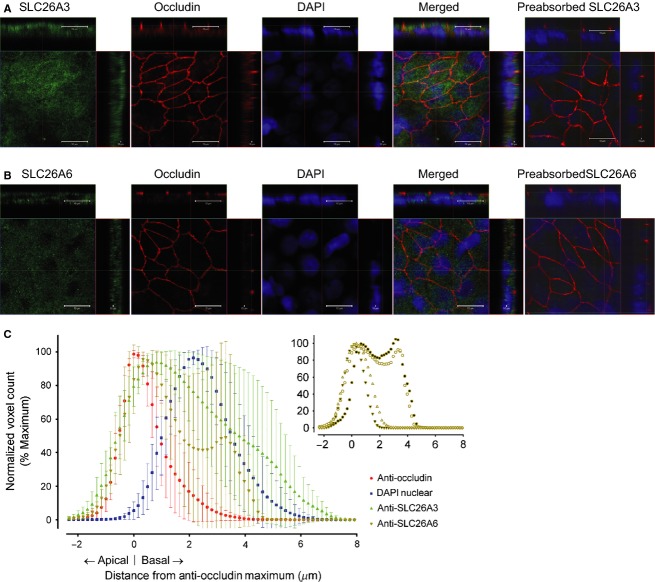
SLC26A3 (A) and SLC26A6 (B) immunoreactivities (green) are observed in primary cultures of 1°PVD cells. Images are from the optical slice with greatest anti-occludin immunoreactivity (red). Top and right side images represent orthogonal projections in the X-Z and Y-Z dimensions, respectively. Orthogonal images show apical location of occludin immunoreactivity. SLC26A3 and SLC26A6 immunoreactivities are greatest at the apical aspect, but diffuse labeling is present throughout the cells. No signal was observed when primary antibodies were preadsorbed. DAPI (blue) was used to demonstrate the nuclei location. White scale bar represents 10 *μ*m. (C) Distribution curves of the mean normalized voxels number (mean ± SD) for occludin, SLC26A3 and SLC26A6 immunoreactivities and for DAPI. Four monolayers from four boars were studied. Inset demonstrates the anti-SLC26A6 labeling for each of the four monolayers demonstrating two distinct labeling profiles.

To determine the distribution of SLC26A3 or SLC26A6 in 1°PVD monolayers, dual immunolabeling (either SLC26A3 or SLC26A6 along with occludin) and confocal microscopy were conducted. Occludin immunoreactivity exhibited a continuous reticular pattern, present near the apical aspect of cells throughout each monolayer (Fig.2). SLC26A3 immunoreactivity was present throughout optical slices in Z-stacks, and orthogonal X-Z and Y-Z projections showed that the highest intensity was at or near the apical aspect of cells (Fig.2A). Although a subset of the observations revealed SLC26A6 immunoreactivity concentrated at the apical region (Fig.2B), an alternative pattern of distribution was also detected and this was further investigated as described subsequently. Specificity of SLC26A3 and SLC26A6 immunohistochemical signals was verified via primary antibody preadsorption (Fig.2A and B).

Detection of apparently distinct patterns of SLC26A3 and SLC26A6 distribution led us to conduct voxel analysis on the outcomes acquired. Within each Z-stack, the slice with the greatest number of voxels positive for occludin was employed as a reference point for distribution analysis. As is shown in Figure2C, the SLC26A3 voxel distribution displays a single peak with its maximum near the apex (0.83 *μ*m toward the basal aspect) of the occludin distribution. A substantial portion of the SLC26A3 immunoreactivity is in or near the apical membrane as there is substantial overlap with occludin immunoreactivity. The SLC26A3 signal is also present throughout the profile of the epithelium. Unlike the SLC26A3 signal, SLC26A6 shows two distinct distribution patterns (Fig.2C inset). Three Z-stacks were examined from each of four cell isolations. A peak in labeling was observed near the apical membrane in all monolayers. A second peak in SLC26A6 labeling was present basal to the DAPI peak in two of the four monolayers that were evaluated. When data from all monolayers are combined, the variation in signal in the basal portion of the cell is quite substantial (Fig.2C). The SLC26A6 signal exhibits a steep decline toward the basal membrane, where it becomes undetectable. The nuclear (DAPI) and SLC26A3 signals extend substantially farther toward the base of the cell when compared to the SLC26A6 signal. These results suggest that SLC26A3 is localized preferentially at or near the apical membrane in all cells whereas SLC26A6 can be observed in two distinct locations within the epithelial cells, at the apical membrane and sometimes near the nucleus.

### SLC26A3 and SLC26A6 immunoreactivities are present in native vas deferens

**Figure 3:**
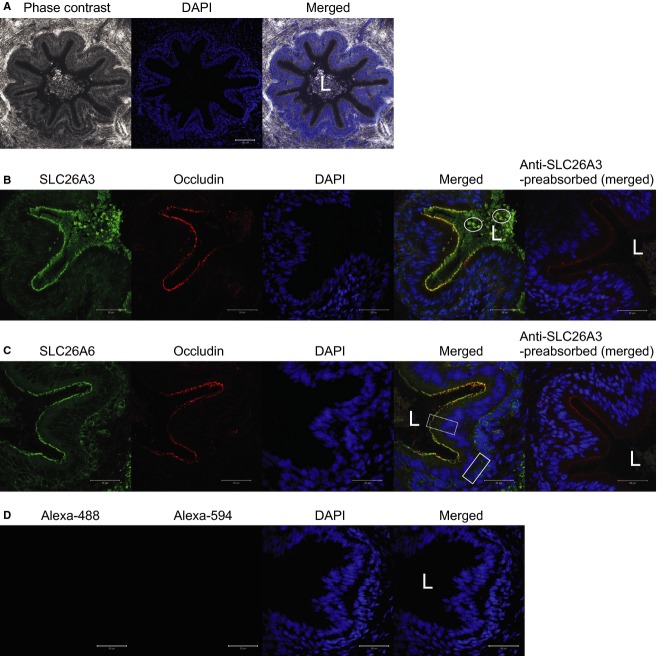
Bicarbonate exchangers SLC26A3 and SLC26A6 are expressed at the apical membrane of cells lining porcine vas deferens. (A) Images from typical transverse sections of intact snap-frozen vas deferens. A lumen (L) is lined by epithelia. Cellular nuclei are labeled in blue (DAPI). Scale bar represents 100 *μ*m. (B and C) Typical patterns of SLC26A3 and SLC26A6 immunoreactivities (green) are shown. Intense SLC26A3 and SLC26A6 immunoreactivities were located at or near the apical aspect of principal cells (dashed-line box frames a few principal cells) throughout proximal and distal segments of porcine vas deferens. SLC26A3 immunoreactivity was intense in sperm heads (circled by ovals). SLC26A6, and to a lesser extent SLC26A3, immunoreactivity was also observed in the area of basal cells (solid-line box frames a portion of this area). No signal was detected when primary antibodies were preadsorbed by their respective immunizing peptides. (D) No signal was detected when primary antibodies were omitted. Scale bar represents 50 *μ*m for panels B, C, and D. Proximal and distal segments from seven animals were studied.

Transverse cryosections of proximal and distal vas deferens segments were analyzed using immunohistochemistry and confocal microscopy. Evidence for the expression of both SLC26A3 and SLC26A6 in epithelial cells lining native porcine vas deferens is provided in Figure3. Phase contrast images combined with DAPI fluorescence (Fig.3A) demonstrate a lumen surrounded by an epithelium that consists primarily of principal and basal cells. Morphological characteristics of the epithelial cells are typical and consistent with previous descriptions (Popovic et al. 1973; Tomaru et al. 1974; Hoffer 1976; Orlandini et al. 1980; Leong and Singh 1990). Phase contrast images demonstrate that the cryosections have well-preserved tissue morphology and an intact epithelium. Principal cells have a distinct apical-lumen border and large apical cytoplasmic space. Images presented in Figure3A clearly show luminal content that exhibits characteristics of sperm including both typical sperm cell morphology and the presence of SLC26A3 immunoreactivity on sperm heads (Chen et al. 2009) (Fig.3B). Luminal contents were present in sections of both the proximal and distal vas deferens. Occludin-specific immunoreactivity was used as a reference point to define the apical aspect of the epithelial cells. SLC26A3 and SLC26A6 immunoreactivities are prominent at or near the apical membrane of principal cells lining the porcine vas deferens (Fig.3B and C, respectively). SLC26A6 immunoreactivity is also observed at areas typically occupied by basal cells. Specificities of the SLC26A3 and SLC26A6 immunoreactivities were demonstrated by the absence of labeling when antibodies were preadsorbed to the respective immunizing peptides (Fig.3). Comparative analysis of SLC26A3 and SLC26A6 immunoreactivity intensities in proximal versus distal vas deferens segments, based on voxel counts, did not reveal any differences between these duct segments. Immunoreactivities detected at basal cells varied widely across the samples analyzed and intensity was quite low in tissues derived from two boars (out of seven examined). Regardless, the simplest interpretation of these observations is that both SLC26A3 and SLC26A6 are highly expressed at or near the apical membrane of virtually all cells lining the porcine vas deferens lumen. Thus, by being in the apical lining, these 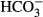 exchanger's activities should contribute to luminal pH modulation.

### Apical biotinylation captures both SLC26A3 and SLC26A6

**Figure 4:**
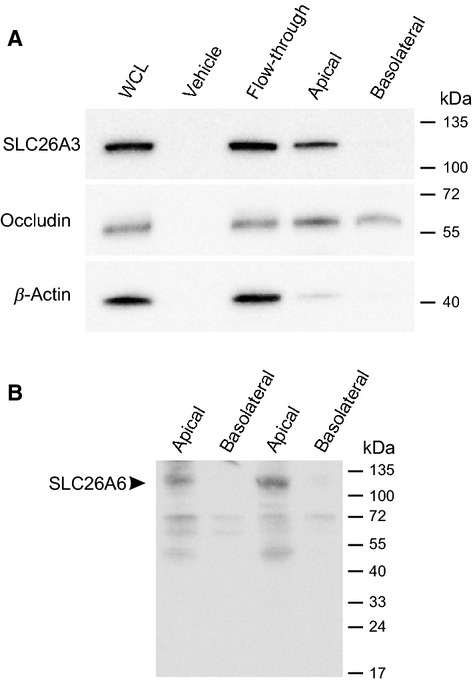
HCO_3_^−^ exchangers SLC26A3 and SLC26A6 are expressed in the apical membrane of 1°PVD cells as indicated by surface biotinylation. WCL, whole-cell lysate; Vehicle, represents WCL from monolayers not exposed to biotin, but subjected to the same protein isolation protocol; Apical or Basolateral indicates the surface exposed to biotin; Flow through represents the first eluate collected from the avidin column and represents the intracellular fraction of protein not accessible to extracellular biotinylation. Positions of standard mobility markers are shown on the right. Images are representative of 4 (A) and 3 (B) boars.

The hypothesis that SLC26A3 and/or SLC26A6 are present in vas deferens apical cell membranes, rather than being sequestered in a sub-apical pool, was tested by conducting surface biotinylation of cultured 1°PVD cells with avidin purification of cell lysates followed by western blot analysis. Results presented in Figure4 show prominent bands of immunoreactivity for SLC26A3, SLC26A6, occludin, and *β*-actin in whole-cell lysates and in the flow through from the avidin columns. Importantly, apical, but not basal, biotinylation produced a prominent band when probed for SLC26A3 or SLC26A6 immunoreactivities. These results suggest that the tight junction protein, occludin, was conjugated to biotin regardless if exposure occurred either from the apical medium or from the basal medium, which included gaining access across the permeable cell culture support. *β*-Actin was expected to be inaccessible to the biotin from either medium and, indeed, virtually no *β*-actin was retained by the avidin column following biotin exposure. The simplest interpretation of these data is that both SLC26A3 and SLC26A6 are present in the apical membrane of 1°PVD cells. Additionally, both 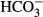 exchangers appear to be present in the cytosol or in a vesicular population where biotin cannot react with them, as their immunoreactivities were detected in flow through (Fig.4).

## Discussion

Results from this study provide molecular evidence demonstrating that 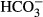 exchangers SLC26A3 and SLC26A6 are expressed by epithelial cells that line the porcine vas deferens. Experiments were carried out using primary cultures of porcine vas deferens epithelial cells to demonstrate the expression of SLC26A3 and SLC26A6 at mRNA and protein levels, along with cryosections of native tissues to demonstrate tissue and cellular localizations of SLC26A3 and SLC26A6 in the native cells lining the intact vas deferens. Notable observations included that: (1) mRNA coding for SLC26A6 is in substantially greater abundance than that coding for SLC26A3; (2) both SLC26A3 and SLC26A6 exhibit their greatest expression at or near the apical membrane while SLC26A6 exhibits, in some cases, an additional peak in expression near the nucleus; (3) both SLC26A3 and SLC26A6 are present in the apical membrane; and (4) the relative and absolute expression of SLC26A3 and SLC26A6 appears to be unchanged over the length of the native vas deferens. The results suggest that principal cells lining the porcine vas deferens are poised to deliver 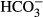 to the duct lumen. This could, in turn, initiate or enhance sperm motility prior to or during ejaculation.

Virtually all cells lining the vas deferens express both SLC26A3 and SLC26A6 immunoreactivity. Likewise, all 1°PVDs tested exhibited immunoreactivity for both epitopes in the apical membrane. These results suggest that a homogenous population of principal cells lines the porcine vas deferens, although two distinct patterns of distribution were observed for SLC26A6 in 1°PVDs. The basis and impact of these distribution patterns remains to be determined. Both human (Hoffer 1976; Paniagua et al. 1982) and rat (Hamilton et al. 1977; Kennedy and Heidger 1979; Andonian and Hermo 1999) vas deferens are reported to have a small number of clear cells (mitochondrial rich cells), dark cells, and pencil cells. The possibility that these cells are present in porcine vas deferens cannot be ruled out based on the observations that are reported. However, data reported here suggest that an overwhelming majority of cells lining the vas deferens express proteins that likely contribute to 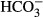 secretion. Previously, we reported that SLC4A4e1b, an electrogenic Na^+^/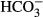 cotransporter, is expressed in basolateral membranes of cultured vas deferens epithelial cells (Carlin et al. 2002) and in PVD9902 cells, a cell line derived from porcine vas deferens (Carlin et al. 2006). Thus, in addition to the previous evidence for a basolateral 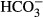 loading mechanism, the current observations document proteins in the apical membrane that can extrude 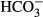 into the luminal compartment.

Cells lining the male reproductive duct modulate the composition of the luminal environment to which sperm are exposed. Luminal pH is a particularly important factor as it is widely appreciated that sperm are exquisitely sensitive to pH (Carr et al. 1985; Brokaw 1987). Luminal pH in the male reproductive duct is typically thought to be mildly acid with the cauda epididymis exhibiting the lowest pH (Levine and Marsh 1971; Carr et al. 1985). It is reported that clear cells present in the epididymis secrete H^+^ to reduce luminal pH, which is thought to promote sperm maturation (Blomqvist et al. 2006) and to provide an amenable storage environment (Au and Wong 1980; Breton et al. 1996, 1999; Brown et al. 1997; Shum et al. 2009). We showed that porcine vas deferens pH may be neutral or slightly alkaline and, importantly, systemic adrenergic agonist administration to anesthetized boars causes rapid and significant increases in luminal pH (Pierucci-Alves et al. 2010). The pH increases we reported are consistent with previous predictions, based on ion transport studies with cultured vas deferens epithelial cells, that the vas deferens could secrete nearly 10^−6^ base equivalents in as little as 15 min (Sedlacek et al. 2001). Such an alkalinization and increase in 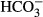 concentration would activate sperm Ca^2+^ channels (Kirichok et al. 2006), K^+^ channels (Navarro et al. 2007), and soluble adenylyl cyclase (Chen et al. 2000), all of which would combine to initiate sperm motility. The activity of SLC26A3 and/or SLC26A6 at the apical membrane of vas deferens epithelial cells, along with SLC4A4e1b in the basolateral membrane, might contribute to the alkalinization that was reported (Pierucci-Alves et al. 2010).

The relationship of SLC26A3 and/or SLC26A6 to net 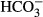 secretion is unclear. Both of these anion exchangers are present in a variety of ductile tissues including salivary glands, intestine, and especially pancreatic ducts. We now add to this list compelling evidence for expression of both transporters throughout the epithelium lining the vas deferens. The mechanism used to accomplish 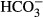 secretion and a cell model defining the relationship of the various transporting components in the vas deferens is not available. Both SLC26A3 and SLC26A6 have been associated with CFTR via binding motif interactions, which are thought to modulate net 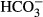 secretion (Ko et al. 2004). The tissue model proposed to account for the secretion of a 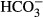 -rich fluid by the pancreas included CFTR functionally coupled to SLC26A6 in the proximal duct and coupled to SLC26A3 in distal portions of the duct. Further, the model required different electrogenic stoichiometries for the two exchangers (Ko et al. 2004). While it has been shown that CFTR is expressed in porcine vas deferens as well as vas deferens epithelia of other species, the results reported here revealed no apparent difference in SLC26 A3 or A6 expression patterns at the proximal and distal vas deferens, which in an adult boar can be separated by 20 cm or more. Thus, the model proposed for the pancreatic duct probably is not applicable to the vas deferens. Although the maximal 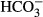 concentration achieved in the vas deferens is unknown, which precludes any predictions regarding a working model to account for net 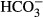 secretion, several inferences can be drawn based on the following clinical observations. First, mutations in CFTR are associated with male infertility; profound mutations are associated with vas deferens atresia and mild mutations are associated with other forms of male infertility (Jakubiczka et al. 1999; Jarzabek et al. 2004). Second, mutations in SLC26A3 are associated with nonobstructive male infertility (Hoglund et al. 2006). Third, no reproductive phenotype has yet been linked to SLC26A6 mutations. If one assumes that vas deferens 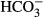 secretion is important for male fertility, then certain conclusions might be drawn. The observations suggest that SLC26A3 and SLC26A6 fulfill different roles in male reproduction as SLC26A6 activity cannot fulfill the role normally performed by SLC26A3 when the latter is mutated. Additionally, SLC26A3 and SLC26A6 are apparently unable to secrete sufficient 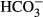 in the absence of CFTR, which may reflect a lack of apical Cl^−^ supplied by CFTR for anion exchange, a lack of Cl^−^ exit from principal cells following anion exchange, a lack of regulation by molecular interaction (e.g., via the STAS domain) (Ko et al. 2004), or perhaps a lack of appropriate trafficking or targeting in the absence of CFTR. CFTR is reportedly permeant to 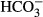 (Tang et al. 2009), but clearly, CFTR cannot fulfill the role of SLC26A3 in 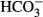 secretion. Taken together, it is logical to conjecture that 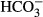 secretion in the male tract that is necessary for fertility is mediated in combination by CFTR and SLC26A3 (and likely SLC26A6).

In conclusion, the 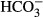/Cl^−^ exchangers SLC26A3 and SLC26A6 are present in high abundance in the apical membrane of virtually all cells lining the porcine vas deferens. Other ion transporters that can contribute to both 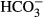 and Cl^−^ secretion are, likewise, present in these cells. The physical and functional relationships between these transporters, however, remain to be determined. Nonetheless, working in concert, this constellation of transporters can account for rapid alkalinization of the duct lumen that would be expected to affect sperm activity and ultimately male fertility.
